# Social Networks and Depression Over Time: The Mediating Role of Social Support and Social Isolation

**DOI:** 10.1093/geroni/igaa057.1312

**Published:** 2020-12-16

**Authors:** Abhijit Visaria, Pildoo Sung, Angelique W M Chan

**Affiliations:** Duke-NUS Medical School, Singapore, Singapore

## Abstract

It is well established that a strong social network is an important factor for successful ageing, specifically for promoting and maintaining psychological wellbeing at older ages. Strong social networks are a source of social support especially at a time of need, and can also help older adults remain connected, active, and engaged in group activities. In this study, we seek to better understand the underlying pathways in the relationship between social networks and depressive symptoms, specifically comparing the extent to which the relationship is mediated by the receipt of material/monetary support, relative to perceived social isolation. We use data from the Panel on Health and Aging of Singaporean Elderly, a nationally-representative study of older Singapore citizens and permanent residents aged 60 years and older in 2009 (N=4990), with two follow-up surveys in 2011 (N=3103) and 2015 (N=1572). We conduct cross-lagged mediation analysis, and control for a number of potential confounders including cognitive function, chronic physical ailments, socioeconomic status, and demographic variables such as age, sex, marital status, and family size. Our preliminary analysis shows that a reciprocal relationship between social networks and depressive symptoms is mediated to a larger extent by social isolation compared to weaker social support.

